# Palliative and supportive care in head and neck cancer: United Kingdom National Multidisciplinary Guidelines

**DOI:** 10.1017/S0022215116000633

**Published:** 2016-05

**Authors:** H Cocks, K Ah-See, M Capel, P Taylor

**Affiliations:** 1 ENT Department, City Hospitals Sunderland, Sunderland, UK; 2 Department of Otolaryngology – Head and Neck Surgery, Aberdeen Royal Infirmary, Aberdeen, UK; 3 George Thomas Hospice Care, Ty George Thomas, Park Road, Whitchurch, Cardiff, UK; 4 St Benedict's Hospice, Sunderland, UK

## Abstract

This is the official guideline endorsed by the specialty associations involved in the care of head and neck cancer patients in the UK. It provides recommendations on the assessments and interventions for this group of patients receiving palliative and supportive care.

**Recommendations:**

• Palliative and supportive care must be multidisciplinary. (G)

• All core team members should have training in advanced communication skills. (G)

• Palliative surgery should be considered in selected cases. (R)

• Hypofractionated or short course radiotherapy should be considered for local pain control and for painful bony metastases. (R)

• All palliative patients should have a functional endoscopic evaluation of swallowing (FEES) assessment of swallow to assess for risk of aspiration. (G)

• Pain relief should be based on the World Health Organization pain ladder. (R)

• Specialist pain management service involvement should be considered early for those with refractory pain. (G)

• Constipation should be avoided by the judicious use of prophylactic laxatives and the correction of systemic causes such as dehydration, hypercalcaemia and hypothyroidism. (G)

• Organic causes of confusion should be identified and corrected where appropriate, failing this, treatment with benzodiazepines or antipsychotics should be considered. (G)

• Patients with symptoms suggestive of spinal metastases or metastatic cord compression must be managed in accordance with the National Institute for Health and Care Excellence guidance. (R)

• Cardiopulmonary resuscitation is inappropriate in the palliative dying patient. (R)

• ‘Do not attempt cardiopulmonary resuscitation’ orders should be completed and discussed with the patient and/or the family unless good reasons exist not to do so where appropriate. This is absolutely necessary when a patient's care is to be managed at home. (G)

## Introduction

Palliative care aims to improve the quality of life (QoL) of patients and their carers facing the problems associated with life threatening illness. This can be achieved by the prevention and relief of suffering, ensuring comfort and dignity, by means of early identification, assessment and management of pain and other, physical, psychosocial and spiritual issues.

Patients with head and neck cancer are a group in whom both specialist palliative and supportive care is especially appropriate whether the treatment intent is curative or not, since the disease and its treatments result in a huge burden of morbidity: short and long term – even lifelong for survivors. In addition to the physical symptoms, these patients often have very significant comorbidities, including tobacco and alcohol dependence, and complex psychosocial issues.

All professionals caring for head and neck cancer patients should assess palliative and supportive care needs in initial treatment planning, and throughout the illness, and be aware when specialist palliative care expertise is needed. This may involve core multidisciplinary team (MDT) members, social workers, psychologists etc. Levels of intervention may involve in-patient, out-patient, day care, home care and telephone advice, from a single, arm's length intervention to a taking over of care. Support provided will need to accommodate any communication impediment. In turn, specialist palliative care practitioners need to be aware of when and how to use palliative interventions such as surgery, radiotherapy (RT) and chemotherapy. All this is best achieved by a high level of integration of services – team working, including the primary care team – and excellent communication, with the ‘key worker’ (usually a specialist nurse) at the centre.

BOX I **MAIN TARGETS FOR PALLIATIVE CARE INTERVENTIONS IN HEAD AND NECK CANCERS**

Medical and surgical treatments

- Pain
- Hydration and nutrition
- Gastrointestinal symptom relief
- Anxiety
- Agitation
- Dysphagia
- Dyspnoea
- Bleeding
- Airway management
- Hypercalcaemia

Holistic, psychosocial and complementary

- Breaking bad news
- Patient aspirations and expectations
- Anxiety
- Counselling
- Psychological support
- Emotional support
- Support groups
- Massage therapy
- Aromatherapy

**Recommendation**

• Palliative and supportive care must be multidisciplinary (G)

## Approaches

Palliative care takes a holistic approach, addressing physical, psychological, social and spiritual needs of the patient, their carers and family (Box I). Interventions which may be appropriate to palliative care include oncological and surgical approaches, drug management, psychological support, Allied Health Professional (AHP) input and complementary therapies. This paper focuses on medical and surgical interventions for physical symptoms, but these should be addressed as part of a wider holistic and multidisciplinary approach, which includes concern with psychosocial and spiritual issues.^1^^–^^3^

Whilst the distinctions between physical and psychosocial symptoms should not be overstated, different interventions will dominate in each category. Drugs, anticancer treatments such as RT, surgery and procedures will dominate in the first category, whilst counselling, honest communication, support groups and complementary therapies will be preferred in the second. This distinction is not clear-cut, however; counselling and honest communication are important parts of pain relief, whilst drugs have a role in the management of symptoms such as anxiety and depression. A well-developed multidisciplinary approach, coupled with an open-minded approach to intervention, is therefore essential.

It is the role of the MDT team to discuss treatment options in all patients. This includes decisions on who should be treated and what is untreatable disease. This is a complex issue and although broad guidelines can be applied each case should be assessed individually. Radical treatment in advanced or recurrent head and neck cancer may be futile and result in poorer QoL, therefore important decisions need to be made at presentation about which treatment pathway to take. The alternative where there is a low chance of cure is a palliative pathway. Palliative treatments include surgical and non-surgical interventions with the intention of slowing disease growth and symptom control, and extending life with focus purely on symptom control.

Effective decision making in the palliative setting is important. The patient and family should adequately understand the diagnosis and prognosis, especially if the trajectory changes due to intervention or disease progression. It should be made clear that symptoms will be identified and treated and patients should be asked if there are any new goals for their treatment since cure is not possible. In other words, the team should not convey a sense of hopelessness simply because the goal is not indefinite survival. Hope can be maintained within the context of the patient's own goals whether they are:

• physical – relief of symptoms
• psychological – fear of distress, suffocation, bleeding or uncontrollable pain at the end of life
• Social – desire to witness a family event, celebrate a birthday or make a trip.

Symptoms should be actively sought and treated in a proactive manner, and it should not be assumed or conveyed that any new symptom is as uncontrollable as the tumour itself. Treatment options should be discussed for the new symptom including those that may not extend life. Although patient choice is central to the treatment options taken, the treating clinician should make recommendations to guide treatment and share the burden of difficult decisions.

**Recommendation**

• All core team members should have training in advanced communication skills (G)

## Symptom control

### Surgical palliation

Incurable end-stage head and neck cancer leads to distressing symptoms. Patients may remain active and self-caring while trying to cope with problems of pain, swallowing, breathing and bleeding. Palliative surgery may be indicated in such cases. Little high-level evidence is available to confirm the surgical benefit; however, descriptive studies support its use in selected cases. Surgery can reduce primary tumour bulk, reduce pain and bleeding, improve swallowing, nutrition and improve and airway (see below). Debulking surgery for advanced neck disease can achieve symptom control, but major resections only rarely offer levels of benefit, which justify the extent of surgical morbidity.

Newer endovascular techniques, including embolisation and vessel stenting, may offer symptom control for bleeding related to major vascular erosion, and these interventions can be considered in patients at high risk of erosion of major vessels.

Acute haemorrhage from carotid ‘blow-out’ (erosion of the carotid vessels) is a distressing end of life event. Whilst occasional success can be achieved with swift surgical intervention, many patients succumb rapidly. In these cases, attempts to reduce the flow of blood with direct pressure while administering appropriate rapid acting sedatives (e.g. benzodiazepines) should be made. Constant verbal support to the patient is a key to help handle anxiety. Do not leave the patient's side.

If surgical intervention is considered inappropriate careful discussion and measured information giving to the patient (if they wish to participate) or family members and carers is essential. This should include the anticipated clinical scenario and an acceptable plan of care should be devised to manage these circumstances. This may include the use of dark towels, anticipatory prescribing, and may influence preferred place of care.

**Recommendations**

• Palliative surgery should be considered in selected cases (R)
• For control of bleeding endovascular stenting or embolisation should be considered (R)

### Non-surgical palliation

#### Radiotherapy

Debate continues around the optimal dosage regimen for palliative RT. Low-level evidence exists for the use of hypofractionation schedules and short course RT. Other protocols such as those described by the Radiation Therapy Oncology Group have also demonstrated benefit. Symptom control can be achieved in up to 80 per cent of selected patients with particular response in terms of pain control. No high-level evidence exists to support one protocol over another, but case series report benefit. Re-irradiation may be offered but may be associated with severe radiation toxicity.

A systematic review of RT for painful bone metastases reports benefit in up to 50 per cent of patients.^4^ There is evidence to support the use of bisphosphonates to aid pain control of bone pain as an additional step once RT and conventional pharmacology has been used. The role of the new monoclonal drugs including RANK – ligand inhibitors (e.g. denosumab) has yet to be elucidated.

#### Chemotherapy

This includes the use of platinum-based agents, 5-fluorouracil and methotrexate, either as monotherapy or in combination with RT and demonstrates benefit in symptom control and QoL measures, but may increase toxicity and hence side effects from the treatment. Careful consideration of the balance between benefit and harm must be made on an individual patient basis. Non-platinum-based agents are reported as conferring symptom control in the selected cases.

#### Future modalities

Future research will include the role of taxanes, e.g. paclitaxel, monoclonal antibodies e.g. cetuximab, newer chemotherapeutic agents, photodynamic therapy and interstitial laser therapy. Descriptive series report some symptom controls using these modalities but without any evidence of improved survival.

**Recommendations**

• Hypofractionated or short-course RT should be considered for local pain control and for painful bony metastases (R)
• Bisphosphonates can be considered for bone pain following RT (R)

### Palliation of dysphagia

Forty per cent of patients with head and neck cancer suffer from dysphagia. This is due to:

• mechanical obstruction
• functional obstruction
• drug induced side effects
• fistula
• pain.

Assessment of the swallow is essential in palliative head and neck patients. It is important to establish whether oral intake is possible and whether it is safe. Aspiration is not uncommon and may be silent in up to 40  per cent of patients, thus the bedside assessment is of limited value. Functional endoscopic evaluation of swallowing (FEES) is straightforward, easily repeatable, portable and can give good information on the aetiology of aspiration as well as feedback to the patient on trials of preventative manoeuvres. It can also be useful in the assessment of ability to deal with different textures and complements information obtained from videofluoroscopy.

Aspiration does not inevitably mean no oral intake. A degree of aspiration may be well tolerated and methods taught to clear the airway after swallowing can be implemented. Similarly certain textures may be better tolerated and the use of thickened fluids can help maintain oral intake. It is important to take into account the patient's wishes and the patient may make an informed choice to continue to swallow despite the potential and real risk of aspiration pneumonia. Quality of life is absolute.

In patients who are unable to swallow, the use of an enteral route via nasogastric tube (NGT) or gastrostomy allows for hydration, nutrition and medication. The type of tube used depends largely on ability to pass an NGT or fashion a gastrostomy, perceived duration of use and patient choice. If enteral nutrition via NGT is likely to extend beyond two to three weeks then gastrostomy should be considered and discussed with the patient.

There exists no clear guidance on when or if it is acceptable to withdraw nutritional support. Patient and family wishes are crucial in this decision process and full consultation is imperative.

Conventional treatments can be helpful in the palliation of swallowing. Surgical debulking either with or without the laser or debrider and RT may help reduce bulk in a hypopharyngeal tumour, dilatation can help in stricture formation and this can be surgical or radiologically guided. Stenting may play a role but often head and neck tumours are too high to accommodate a stent comfortably and without impacting on other functions.

**Recommendations**

• All palliative patients should have a FEES assessment of swallow to assess for risk of aspiration (G)
• Establishment of enteral feeding must be considered early in patients who are unable to maintain their intake orally (G)

### Palliation of the airway

Where there is airway compromise it is common practice to consider a tracheostomy. However, it may be possible to avoid tracheostomy in some cases if the consideration is given to surgical debulking techniques. This is dependent on local expertise and equipment.^5^

Sometimes avoiding surgical intervention is the most appropriate course of action, for example, a patient with a tracheal tumour that has been repeatedly debulked, and has received palliative RT, is not a candidate for stenting. There will come a time when the airway compromise will be life threatening. A tracheostomy may not be an option in this instance. In such instances opioids for dyspnoea in addition to palliative sedation and reduction of secretions can support a patient in a terminal event.

These situations are difficult and information should be imparted to the patient sensitively. In the situation where the patient wants to fully discuss the anticipated scenario a sense of control can be restored to them by discussing what interventions can be undertaken pharmacologically to avoid any distress. If the patient does not want to participate in the discussion this should be documented and discussed with family and/or carers. This situation may influence the preferred place of care. To have the patient and the family prepared for the event is paramount. They must know what will be in place to prevent the dyspnoea and anxiety associated with such a situation and the patient must be comfortable to the end.

If a tracheostomy is indicated local protocols should exist or be developed to help the patient, the family and community staff manage tracheostomy wound care along with maintenance of a clean secure tube. Heat moisture exchange and voicing attachments may be used to aid patient communication.

### Pain

Pain is very common, affecting most patients at any stage. It may be disease or treatment related, either acute and/or immediate or persistent and/or lifelong. Pain occurring after a long, pain free interval is likely to be recurrent disease. Assessment must take account of the presence of ‘total pain’ i.e. physical, spiritual, psychological and social elements. The three major pain types are all encountered – somatic, visceral and, particularly difficult, neuropathic.

Analgesic use is best based on the World Health Organization (WHO) ‘pain ladder’ (Box II) with three steps of increasing potency, and used depending on pain severity and response. The severity of the pain dictates the strength of the analgesic and the pathophysiology dictates the adjuvant used.

BOX II **WHO PAIN LADDER**

Paracetamol ± non-steroidal anti-inflammatory drug ± adjuvant

Weak opioid (codeine or tramadol) + step 1 drugs

Strong opioid replacing the weak + step 1 drugs

The choice of formulation depends on whether the patient can swallow, is vomiting, or has a nasogastric (NG) or gastrostomy tube in situ.

#### Somatic pain

Morphine remains the first choice strong opioid, other than perhaps in renal impairment when an alternative is preferred. It is initiated by titrating immediate release morphine oral solution or tablet (e.g. Oramorph™ solution or Sevredol™ tablet). Once responsiveness and dosage are known, then sustained release preparations are used, with immediate release doses for breakthrough at a sixth of the 24 hour sustained release dosage. If the patient can swallow, then sustained release tablets (e.g. MST Continus™) or capsules (e.g. Zomorph™) can be used. If a tube is in place then a morphine suspension (e.g. MST suspension™) or opened capsules (e.g. Zomorph™) can be used. If this is not feasible, usually because of vomiting, then a subcutaneous (SC) infusion of morphine or diamorphine can be used, with SC doses for breakthrough. Diamorphine is preferred since it is more soluble and can be used in much smaller volumes.

Transdermal preparations of fentanyl have theoretical and practical attractions for stable background pain as an alternative, particularly if there is morphine intolerance (e.g. sedation and dysphoria) or there is renal failure. For breakthrough pain, oral opioids can still be used. Alternatively, new preparations of buccal, sublingual or intranasal fentanyl may have a role in specific situations, with supervision of a specialist service.

Oxycodone can be an alternative to morphine where there is intolerance, particularly dysphoria; there is an immediate release solution and injection, but there is only a tablet form of sustained release oral preparation, limiting its use where swallowing is compromised. Hydromorphone is not useful orally where swallowing is impossible, both immediate and sustained release being capsules, but it may be injected. Methadone in liquid form can be very useful, being rapid in onset and long acting because of its half-life; it is best used by specialists as it can accumulate.

#### Neuropathic pain

This is very common both as a presenting feature of the disease and a result of treatment, particularly radiation. The drugs used can be referred to as adjuvants.

• A tricyclic antidepressant, most usually amitriptyline is available as tablet and liquid.
• Anticonvulsants such as gabapentin and pregabalin are the most used, available only as tablets or capsules unless through special arrangements with a pharmacy. Gabapentin can be opened and administered via the gastrostomy tube.
• Carbamazepine is an alternative and is available both as tablet, liquid and even suppositories. Sodium valproate is also available as a liquid preparation.

First line would be either antidepressant or anticonvulsant titrated to maximum dose tolerated (usually added to a conventional analgesic): second line would be to use both.

Some advocate corticosteroids (e.g. dexamethasone 8–16 mg daily) as first line for acute neuropathic pain where there is felt to be a significant inflammatory component. Appetite stimulation limits use if dysphagia is a concomitant feature. It is not for chronic or predictably long-term pain. Clonazepam is occasionally useful. Methadone and ketamine are useful, but only in specialist settings.

#### Visceral pain

Treatment depends on the cause, titrating analgesics and using the pain ladder. If the pain is poorly sensitive to opioids, adjuvants should be considered early, for example pain due to metastatic disease in the liver or nerve compression may be eased with Dexamethasone (4–8 mg daily).

Judicious use of all these drugs is best achieved by seeking advice from the specialist palliative care service whenever there is concern. Interventional pain techniques can be very effective where systemic treatments fail or if the patient is intolerant of the significant doses of combination analgesics.

#### Mucosal pain

This can be due to treatment, infection or tumour. Treatment of infection such as candida or herpes is essential. Useful additional topical agents include sulcralfate, benzydamine, chlorhexidine, steroids and topical local anaesthetics such as lignocaine preparations. Coating measures including bioadherent oral gel may be preferred by the individual patient.

**Recommendations**

• Pain relief should be based on the WHO pain ladder (R)
• Specialist pain management service involvement should be considered early for those with refractory pain (G)

### Nausea and vomiting

The approach must take an account of the large number of patients who are enterally fed. Even with this there is often a need for injectable anti-emetics – subcutaneous (SC) boluses or continuous infusions, at least until initial control is established.

Enteral feeding poses its own challenge, and prokinetic drugs such as metoclopramide (tablet, oral solution or injection) or domperidone (tablet, suspension or suppository) may be needed to ensure best function.

Otherwise the approach is similar to that in general use. Remember the practical issue of providing a large bowl, tissues and water for the patient and be prepared to rehydrate using IV or SC fluids if appropriate.

### Constipation

Constipation develops in half of patients who are terminally ill with cancer admitted to a hospice. In addition, it is common during treatment in many patients. This is due to dehydration, reduced physical activity and the use of constipating drugs, particularly opioids and anticholinergic medication. Laxatives should be initiated once opioid medication is prescribed. Hypercalcaemia and hypothyroidism are other causes, which may be overlooked.

The principle of treatment is avoidance and early recognition. Enquiry should be made on patient contact. Laxative agents include stimulants such as bisacodyl and senna and softeners such as lactulose, magnesium hydroxide and docusate. Polyethylene glycol preparations including movicol and laxido are commonly used. These should be used prophylactically. If constipation develops it can lead to nausea and vomiting and in the severe situation pseudobstruction. If rectal examination reveals hard stool then the use of suppositories and enemas can be helpful. Ultimately, a manual evacuation may be necessary.

**Recommendation**

• Constipation should be avoided by the judicious use of prophylactic laxatives and the correction of systemic causes such as dehydration, hypercalcaemia and hypothyroidism (G)

### Confusion and agitation

It is important to distinguish anxiety (unsettled, frightened, panic) from confusion, particularly delirium. Confusion is common, affecting up to 75 per cent of cancer patients at some stage. Many head and neck patients have a history of heavy alcohol (and tobacco) consumption, predisposing them to the effects of withdrawal, and given that cancer is more commonly seen in old age; then cognitive impairment is not uncommon.

Benzodiazepines are the mainstay of pharmacological treatment of anxiety. Diazepam can be given orally, via a tube in liquid form, or by injection intravenously. Lorazepam can be swallowed or a tablet dissolved sublingually. If injections and/or infusions are needed, midazolam is preferred, as it can be given subcutaneously (most common route) or intravenously when almost immediate effect is needed. The key limiting factor, however, is rapidly developing tolerance; benzodiazepines are useful for short-term management of episodes of anxiety, but are limited where anxiety is pre-existing and established.

Delirium as a cause of confusion can be related to a number of organic causes – infection, dehydration, metabolic disturbance, respiratory failure, urinary retention, constipation, brain metastases, etc. Administered drugs are common causes, particularly opioids and drug withdrawal (see above). While treatment has to be aimed at the cause, symptom management is required in the short term. While benzodiazepines have a role, indeed a specific indication in drug withdrawal, most often delirium is better managed using haloperidol (as tablet, liquid or injection, including SC) or levomepromazine (as tablet or injection) where sedation is needed in managing paranoia etc.

In some cases, particularly for irreversible agitation or delirium in a dying patient, benzodiazepines and antipsychotics need to be combined and are often administered using a syringe driver.

**Recommendation**

• Organic causes of confusion should be identified and corrected where appropriate, failing this, treatment with benzodiazepines or antipsychotics should be considered (G)

### Secretions

Although xerostomia is common in these patients, excess secretions and/or the inability to swallow or otherwise clear secretions is often troublesome. Physically the use of suction either by carer or the patient is often helpful.

There are three widely used antimuscarinic drugs.

• Hyoscine hydrobomide (scopolamine) is available as a transdermal patch, oral or sublingual tablet and is commonly used; however, it has central as well as peripheral actions and (unpredictable) sedation and/or confusion can result.
• Hyoscine butylbromide, which is not central nervous system active, but equally effective peripherally, and is arguably the drug of choice. It is available as a tablet, though often ineffective by that route; hence SC use may be preferred.
• Glycopyrronium, which is similarly peripherally active, and is most often given subcutaneously. A liquid form can be prepared but efficacy is unpredictable.
• Excess secretions at the end of life are treated similarly, but the evidence in a Cochrane review suggests they are of very limited benefit. Established practice accepts SC preparations of anticholinergic medication are available for use to support this end of life phase. Timely management is a key here; if secretions develop, then regular or continuous antisecretory drugs should be started as soon as practical, rather than relying on PRN drugs.

### Steroids

As with other cancers, corticosteroids are widely used. Dexamethasone (Table I) is the most used, because of its potency, relative lack of mineralocorticoid properties, and wide range of formulations (water soluble tablets, solution, and injection, SC or intravenous).^6^

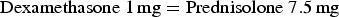

**Table I:** Indications and dosage for steroid (dexamethasone) use

Appetite, energy and wellbeing	4 mg initially
Adjuvant analgesic	8–16 mg initially
Anti-emetic	See above
Spinal cord compression	See NICE guidelines^7^
Tumour oedema (e.g. tracheal compression, superior vena cava obstruction)	8–16 mg initially

Long-term use also requires that attention be paid to bone mineral density, and bisphosphonates, and calcium and/or vitamin D supplements are indicated.

BOX III **SPINAL METASTASES**

Type of associated pain

Pain in spine (new or progressive)

Spinal pain aggravated by straining

Localised spinal tenderness

Pain in spine at night preventing sleep

Neurological symptoms and signs

Radicular pain

Limb weakness

Difficulty walking

Sensory loss

Bladder or bowel dysfunction

Signs of caudal equina/spinal cord compression

If used for any length of time patients must carry a ‘steroid card’, keep it up to date, and be aware of the advice on it, i.e. to increase the dose when there is intercurrent illness or other stressor; and the need to reduce very gradually if used for more than three to four weeks – including at the end of life. Some advise that steroids given for poor appetite or fatigue can be discontinued then. This puts the patient at risk of steroid insufficiency, an unnecessary symptom burden even at that stage, and dexamethasone can be given in small volumes subcutaneously once daily, as part of end of life care if appropriate.

### Spinal metastases

The incidence of spinal metastases in head and neck squamous cell carcinoma is reported to be less than 2 per cent; however, it is more common in thyroid cancer (2–13  per cent). The most important factor in determining outcome is neurological status prior to treatment. Due to the devastating neurological sequelae of spinal cord or cauda equina compression early recognition (Box III) and action is essential and consideration that symptoms may be suggestive of spinal metastatic disease is the first step.^7^

Neurological symptoms and signs should be assessed and a magnetic resonance imaging of the whole spine obtained. This is an oncological emergency and steroids should be commenced while investigations or admission are arranged. Treatment depends on findings and includes steroids, surgical stabilisation and RT. Clear guidelines on diagnosis and management have been published by National Institute for Health and Care Excellence (NICE) and the readers should familiarise themselves with these.^7^

**Recommendation**

• Patients with symptoms suggestive of spinal metastases or metastatic cord compression must be managed in accordance with the NICE guidance (R)

## Care of the dying

Care of the dying is an important part of good palliative care. Dying patients may have significant and rapidly changing symptoms, together with a recognition that no further active intervention is appropriate. For these reasons, timely assessment, regular review and confident symptom control are essential. In addition, this is an important time for loved ones; as noted by Dame Cicely Saunders, ‘How people die remains in the memories of those who live on’. Ongoing sensitive and honest communication, coupled with sensible and proactive decision-making are therefore essential.^8^

Reversible causes for a patient's deterioration should be considered and may be acted upon depending upon earlier discussions, clinical acumen and based on the best interests of the patient. The physical changes preceding death generally include decreasing mobility, decreasing level of consciousness and interaction, minimal intake, progressing to no oral intake, decreasing urine output, haemodynamic deterioration and changes in respiratory pattern. Recognising death is imminent, the doctor may lead multiprofessional decision making and communication ensuring the patient (if appropriate) and families or carers understand the expected trajectory.

The patient's values and preferences should be upheld where possible, these may include rapid discharge to enable the patient to die in the place of their choice, or enable their family to stay with them if in in-patient settings. Any religious, spiritual or cultural preferences should be identified.

The Liverpool Care Pathway (LCP) was a protocol developed at the Marie Curie Institute Liverpool, and in use in the UK between 1997 and 2014. Concerns about the use of the pathway were raised in the press, and a subsequent government review was undertaken. Whilst recognising both good and bad outcomes arising from the use of the pathway, the ultimate recommendation of the review body was that the LCP be withdrawn. Current approach is based on this framework but using a more individualised and tailored care plan. Such plans are currently subject to local variation but can be used in all care settings including patient homes. National guidance is being developed following consultation.

A key role of the doctor is to recognise that death is imminent, and, as recommended in the government review, the patient's senior clinician has a vital role in this decision in the MDT. Recognition of dying should prompt a thorough review of all care and interventions, with unnecessary medication being stopped, and essential medication continued, usually by SC infusions and boluses. In the head and neck patient, the frequent presence of NG and gastrostomy tubes allows continued use of some medications which would otherwise be impossible to administer.

It is important to highlight that recognising dying does not automatically lead to discontinuing any such interventions; only that their role in improving symptoms should be assessed.

Whilst nutrition is usually inappropriate in dying patients neither SC nor intravenous fluid is necessarily ruled out – although the benefits can be, indeed often are very limited. Enteral tubes provide a further option for those patients.

Sensitive discussion with the patient (if appropriate) and family or carers should be initiated to dispel any concerns held and agree a plan appropriate to the individual which may require modification depending upon the timescale and symptoms observed. A further vital aspect of end of life care, recognised both in the LCP and the review, is the need for regular multiprofessional assessment, and the possibility that patients may improve, for whatever reason, and hence the management plan be changed.

Whilst an individualised approach is vital for dying patients, certain symptoms are common enough to warrant ‘anticipatory prescribing’. The four major symptoms for which this is appropriate are:

• pain
• nausea and vomiting
• agitation
• excess secretions.

The choice of drugs used is left to individual units and must be individualised further for some patients. For most purposes:

• analgesia – diamorphine or morphine
• anti-emetic – haloperidol or levomepromazine
• agitation – midazolam and/or levomepromazine or haloperidol
• antisecretory – hyoscine, either butyl or hydrobromide.

Common reasons for modifying the drugs of choice include poor tolerance of previous drugs, cases where other drugs have an already-established role, clinical contra-indications or renal failure. Fortunately, all the commonly needed drugs can be given subcutaneously, and feeding tubes increase the available options. Areas which require ongoing monitoring and vigilance include mouth care, tracheostomy and wound care, pressure areas, and continence.

**Recommendation**

• All patients at the end of life should have anticipatory medication available to palliate common symptoms and should have an individualised care plan (G)

## Do not attempt resuscitation (DNAR) (cardiopulmonary resuscitation (CPR))

This is a subject of such wide clinical and ethical complexity (Box IV and V) that it is not possible to offer more than a few thoughts on the main points. Such a decision applies ONLY to the state of cardiopulmonary arrest – it does not imply withholding other treatments, including other ‘resuscitation’ measures (e.g. reinserting a dislodged tracheostomy tube).

BOX IV **FUNDAMENTAL ETHICAL PRINCIPLES**

Respect for autonomy

Beneficence

Non-maleficence

Justice

BOX V **RELEVANT ARTICLES OF HUMAN RIGHTS ACT**

The right to life

Freedom from inhuman or degrading treatment

The right to privacy

Freedom of expression and to be informed

Freedom from discrimination

When considering palliative and end of life care, one specific area for consideration is that of CPR. Ultimately, any decisions made around CPR should be undertaken in advance. In the event of a cardiac arrest, and where no such decisions have been made in advance, the default position is to perform CPR. In some cases, even in patients with incurable disease, this is appropriate. In the dying patient, however, or in cases where the chances of CPR succeeding are remote, then CPR adds no benefit to patient care. In such cases, a ‘Do not attempt cardiopulmonary resuscitation’ (DNACPR) order should be completed.

There exists a number of issues regarding DNACPR decisions, outlined in national guidance issued by the British Medical Association (BMA), Royal College of Nursing (RCN) and Resuscitation Council (RC), and recently examined in a Court of Appeal Judgement. Two key points stand out – the decision-making around CPR, and the discussion around such decisions. The current BMA, RCN and RC guidance is summarised here, but may be subject to review in the coming months.

### Decisions around CPR

Where a cardiac arrest is a significant possibility, where CPR has a reasonable chance of success, and where no advance decisions have been made with respect to resuscitation, then CPR should be attempted. Examples of such cases include acute reversible illnesses or treatable arrhythmias. Similarly, if a cardiac arrest is unlikely, then CPR should be attempted if it occurs. Examples here include the otherwise healthy person admitted with a relatively minor illness or an out-of-hospital arrest in public. A presumption of patient consent exists here, and it is not relevant to discuss in advance unless requested (and in such a case, patient wish should be respected). Whilst this is applicable to many hospital patients, it is less relevant to palliative care patients, in whom life-threatening events are more likely, and CPR is less likely to succeed.

At the other extreme, where a patient is dying and no reversible causes for their condition exist, then CPR is inappropriate. In this context, cardiac arrest may be viewed as the final event in the process of natural death. Nevertheless, whilst the clinical decision may be clear, serious consideration needs to be given to discussion with the patient and family; this is covered in the section ‘Discussing CPR decisions’, below.

In many cases, including in palliative care, the benefits and burdens of CPR are less clear-cut. For example, in a patient with an ultimately palliative diagnosis but who is otherwise active and well, there is a small chance that CPR in the event of a cardiac arrest may succeed. It is beyond the remit of this work to outline factors that count for and against this. In such cases, the preferences of patients (or those delegated to make decisions on their behalf) are pivotal.

### Discussing CPR decisions

As outlined above, discussing CPR decisions is not relevant in a large proportion of hospital patients, as presumption of consent exists. This section is concerned with those cases where cardiac arrest is a realistic possibility.

#### Where CPR would not succeed

In cases where it has been determined clinically that CPR has no realistic chance of success, the decision rests with the medical team. Any discussion revolves around sensitively informing the patient (and/or any person delegated to be involved in such discussions) of the decision that has been made. Difficulties here arise where the patient or delegated person objects to the decision. In such cases, seeking a second opinion is good practice. It is usually possible to work through such disagreements with time and sensitive communication.

In some such cases, the cited guidance allows for DNACPR decisions not to be discussed with the patient or their delegated decision-maker. This applies to situations where the treating team have strong reason to believe that such discussions will cause significant distress or where the patient has asked not to be involved in such discussions. Citing risk of distress should not be undertaken lightly; any such judgement should be carefully documented and backed up with evidence – such decisions have been challenged in court.

It is important to reinforce that the clarity of the decision is not a factor in considering whether to discuss a DNACPR order. Even where CPR has no chance of success, serious consideration should still be given to discussion.

#### Unclear benefits/burdens: a person with capacity

A competent patient can decline CPR and a DNACPR document can be completed based solely on this decision, provided the clinician completing the document is satisfied that the patient has capacity for the decision and understands it.

Whilst a competent patient may decline CPR, they may not insist on receiving CPR in the event that they suffer a cardiac arrest, if it is deemed that CPR would not succeed. Where there is a possibility of success, eliciting and respecting the patient's wish is crucial. Such discussions should be handled sensitively, and the patient given the opportunity to consider the discussion and invite family members/carers to support them.

There are further subtleties to these decisions, but such discussion is outside the remit of this work. Examples include a patient refusing discussion, or a patient delegating a decision to healthcare professionals. Current professional guidance is helpful in working through these situations.^9^^,^^10^

#### Unclear benefits/burdens: a person with recent loss of capacity

If the patient has recently lost capacity for such decisions, some questions need to be asked:

• Have they previously discussed and agreed to a DNACPR?
• Have they made some other form of advanced decision to refuse treatment/living will?
• Have they been party to ‘Advance Care Planning’?
• If so, are the circumstances those previously envisaged?

It could then be seen as reasonable to let this inform the current decision. It is also important to know whether the patient, when competent, appointed someone with lasting power of attorney under the terms of the Mental Capacity Act, 2005 – in which case this person should be approached, bearing in mind that they, no more than the patient, can insist on treatment, only decline it – *see above*.

#### Unclear benefits/burdens: a person with longstanding loss of capacity

If the patient has a longstanding loss of capacity, then the decision is left to the doctor(s) and other members of the team to act in the patient's best interest, in accordance with the provisions of the Mental Capacity Act. Where available, family, next of kin and carers can be asked if they are aware of any opinions expressed previously by the patient, etc. – again noting that they cannot actually make the decision, only inform the process. In situations where the patient is alone then under the Mental Capacity Act one must involve Independent Mental Capacity Advocate to contribute to the decision- making process.

### Further considerations

It is not possible to cover all eventualities for these decisions, and professional guidance exists and should be followed. Two further issues warrant discussion, however; managing unresolved disagreements and transfer to the home environment.

Despite the emotive nature of the subject and complexity of decisions, it is usually possible to work through DNACPR decisions to the agreement of the patient, their loved ones and the clinical team. As described above, a second opinion can be helpful in resolving a disagreement. Occasionally no agreement can be reached between doctor, the team, the patient and those close to the patient. In extreme cases, particularly where the patient lacks capacity, legal advice may be required and consideration given to more formal measures such as the involvement of the Court of Protection.

A further point to highlight is the transfer of DNACPR decisions to the home environment. In such context, the patient and their family/carers are responsible for the documentation and, as such, are able to ignore or withhold it if they wish. For this reason, clear communication and agreement in advance are vital.

**Recommendations**

• Cardiopulmonary resuscitation is inappropriate in the palliative dying patient (R)
• ‘Do not attempt cardiopulmonary resuscitation’ orders should be completed and discussed with the patient and/or the family unless good reasons exist not to do so where appropriate. This is absolutely necessary when a patient's care is to be managed at home (G)

### Key points

• Palliative care takes an holistic approach addressing physical,psychological, social and spiritual needs of the patient,their carers and family
• Symptoms should be actively sought and treated in a pro active manner by the multidisciplinary team
• Pain is very common, affecting most patients at some point and maybe disease or treatment related.
• Constipation develops in half of patients who are terminally ill with cancer admitted to hospice
• Confusion can affect up to 75% of cancer patients at some stage.
• Spinal metastases should be considered where there is new or progressive back pain and investigated pro actively
• A key role of the doctor is to recognise when death is imminent and should prompt a through review of all care and interventions with unnecessary medication being stopped.
